# Impacts of Black Soldier Fly (*Hermetia illucens*) Larval Meal on Intestinal Histopathology and Microbiome Responses in Hybrid Grouper (*Epinephelus fuscoguttatus* ♀ × *E. lanceolatus* ♂): A Comprehensive Analysis

**DOI:** 10.3390/ani14243596

**Published:** 2024-12-12

**Authors:** Yan Chen, Jun Ma, Yoong-Soon Yong, Yonggan Chen, Bing Chen, Junming Cao, Kai Peng, Guaxia Wang, Hai Huang, Jiun-Yan Loh

**Affiliations:** 1 Yazhou Bay Innovation Institute, Hainan Tropical Ocean University, Sanya 572024, China; cy969398hn@126.com (Y.C.);; 2 Hainan Key Laboratory for Conservation and Utilization of Tropical Marine Fishery Resources, Sanya 572004, China; 3 Key Laboratory of Utilization and Conservation for Tropical Marine Bioresources, Hainan Tropical Ocean University, Ministry of Education, Sanya 572022, China; 4 R&D Quality Department, Osmosis Nutrition Sdn Bhd, Bandar Nilai Utama, Nilai 71800, Negeri Sembilan, Malaysia; yongys@osmosis.com.my; 5 Institute of Animal Science, Guangdong Academy of Agricultural Sciences, Collaborative Innovation Center of Aquatic Sciences, Key Laboratory of Animal Nutrition and Feed Science in South China, Ministry of Agriculture and Rural Affairs, Guangdong Provincial Key Laboratory of Animal Breeding and Nutrition, Guangzhou 510640, China; 6 Tropical Futures Institute, James Cook University Singapore, 149 Sims Drive, Singapore 387380, Singapore

**Keywords:** black soldier fly, enzymes, histopathology, hybrid grouper, intestinal microbiota, sustainable aquaculture

## Abstract

This study examined the diversity and responses of intestinal microbiota in hybrid grouper (*Epinephelus fuscoguttatus* ♀ × *E. lanceolatus* ♂) fed diets with varying levels of fishmeal replaced by black soldier fly larvae (BSFL). The 10% BSFL substitution (BSFL10) group showed the highest levels of trypsin and amylase. Substituting fishmeal with 30% and 50% BSFL weakened the intestinal wall, resulting in vacuoles, sparse striatal boundaries, and fewer villi. Microbiota diversity, measured through Shannon’s index, was higher in the BSFL10 and BSFL50 groups than in the control. 16S rRNA amplicon data revealed the dominance of Firmicutes, Proteobacteria, Bacteroidetes, Spirochaetota, and Verrucomicrobia phyla. The BSFL-replacement groups showed an increase in Proteobacteria, Bacteroidetes, and Spirochaetota compared to the control, but fewer Firmicutes. PICRUSt analysis indicated significant alterations in microbial function, particularly enhanced protein, carbohydrate, lipid, and energy metabolisms in the BSFL-fed group. Substituting 10% fishmeal with BSFL enhanced nutrient metabolism and gut microbiota in juvenile hybrid grouper. Further research is needed to explore factors affecting the efficacy of insect feed as a sustainable aquaculture diet.

## Simple Summary

Fishmeal is commonly used in aquafeed manufacturing. However, the global market price of fishmeal is highly fluctuating, and it is not the most sustainable solution. Therefore, the aquafeed industry is often exploring alternative protein sources such as insect meal to replace fishmeal. Our study involved testing diets where 10%, 30%, and 50% of the fishmeal was replaced with black soldier fly larvae to observe how this affects digestion and gut bacteria diversity in hybrid grouper. The dietary group with 10% substitution showed higher levels of enzymes that help break down food and absorb nutrients effectively. In contrast, the diets with 30% and 50% replacements showed signs of weakening in the intestinal walls, which could negatively affect nutrient absorption. Moreover, the variety of gut bacteria in the groups with 10% and 50% replacement was greater, with modifications observed in the biological functions associated with energy and nutrient processing. Our results suggest that replacing some fishmeal with black soldier fly larval meal could potentially improve gut health and promote the development of sustainable aquaculture diets.

## 1. Introduction

According to the FAO [1], global fish production is projected to reach 204 million tons by 2030, a 15% increase compared to 2018. This growth rate is about half of what was observed in the previous decade. World fish consumption per capita is expected to rise by 5% from 2018, reaching 21.5 kg in 2030. In 2018, global fish production was estimated at approximately 179 million tons, with a total sales value of around $401 billion. China is the leading global aquaculture producer, accounting for about 58% of total production in 2018. One notable demersal species in this country is the hybrid grouper (*Epinephelus fuscoguttatus* ♀ × *E. lanceolatus* ♂), which is highly valued and cultivated in tropical and subtropical waters worldwide, particularly along the coastlines of Southeast Asia and the South China Sea [2,3].

Fishmeal (FM) is a valuable component in aquaculture, poultry, and livestock feeds due to its high protein content, essential amino acids, minerals, and omega-3 fatty acids. However, concerns have been raised about the economic, environmental, and social sustainability of FM in feed formulations. Research is being conducted on alternative protein sources to improve sustainability in fish farming [4,5]. Plant protein sources, such as soybean meal, soybean protein concentrate, peanut meal, and rapeseed meal, have effectively replaced FM in aquaculture diets in recent years [6,7]. Protein utilization in marine fish varies depending on the species, and grouper, in particular, has unique nutritional requirements. The intestinal flora plays a vital role in maintaining the health of the organism by affecting nutrient absorption, preventing pathogens, and promoting immune defense [8,9,10].

Plant-based diets contain indigestible components, such as non-starch polysaccharides (NSPs) [11], along with anti-nutrients like protease inhibitors and lectins in soybeans, glucosinolates in canola, and gossypol in cottonseed flours. Consequently, using these plant protein sources in fish feed has been linked to reduced digestive efficiency, decreased nutrient absorption [12], and alterations in gut microbiota balance [13,14]. Various alternative protein sources, including insect protein from black soldier fly larvae meal, silkworm chrysalis meal, *Gryllodes sigillatus* meal, and *Blatta lateralis* meal, are rich in crude protein and fat, as well as antimicrobial peptides, chitin, and prebiotics [15]. These components can function as ingredients that enhance gut microbiota and immune responses [16,17,18,19,20,21,22].

More than 150 insect species are recognized as sustainable protein sources with adequate nutritional value for animal production in the European and Mediterranean Plant Protection Organization (EPPO) area and North America. Insects provide a protein-rich component of feed that offers promising possibilities for animal nutrition [16]. The use of insects as feed is seen as a significant advancement in animal nutrition [17,23,24]. Among these species, the black soldier fly (*Hermetia illucens*) has a short larval phase, high fertility, and exceptional waste conversion rates. Thus, the inclusion of black soldier fly larval (BSFL) meal in diets has been extensively studied across many fish species. BSFL has a well-balanced profile of amino acids and fatty acids, making it suitable for aquaculture feed formulations [17,25]. Dietary supplementation with BSFL meal can serve as a feasible alternative to FM, comprising 20% to 100% in various fish species, including yellow catfish (*Pelteobagrus fulvidraco*) [26], Jian carp (*Cyprinus carpio* var. Jian) [27], rainbow trout (*Oncorhynchus mykiss*) [28], juvenile turbot (*Psetta maxima*) [29], Atlantic salmon (*Salmo salar* L.) [30], African catfish (*Clarias gariepinus*) [31], hybrid tilapia (*Oreochromis* sp.) [32], European seabass (*Dicentrarchus labrax*) [33,34], red sea bream [35], and Siberian sturgeon [19].

In our previous study, including BSFL in fish diets by replacing FM at levels ranging from 10% to 50% had significantly different effects on fish growth [19]. Based on our results, we recommend an optimal replacement level of BSFL for marine fish of up to 20%. However, assessing the impacts of a feed ingredient solely based on growth performance is insufficient to understand the underlying mechanisms. To date, only a limited number of studies have investigated the effects of substituting FM with varying levels of BSFL in farmed fish species through the characterization of intestinal microbiota composition and histopathology [19].

The gut microbiota plays a crucial role in normal gut function, immunological development, lipid metabolism, and energy balance, which in turn influence host development and physiology [36,37,38,39]. Dietary components significantly impact the gut microbiota, as different diets can selectively promote or suppress the growth of certain microorganism clades, which in turn affect the host [19].

To evaluate the impact of BSFL meal on fish gut microbiota, we used a broader range of BSFL inclusion (e.g., 10%, 30%, and 50%) to understand the significant changes that occur across a different rate of BSFL inclusion. This is important to characterize the interactions between the host and its gut microbiota through metagenomic analyses [40]. Despite the beneficial effects of BSFL meal on the growth of some marine species, its impact on hybrid grouper gut health, function, and microbiota remains unclear. Therefore, this study aims to investigate the effects of partially substituting FM with BSFL meal, using intestinal histopathology and gut microbial metagenomics. This study represents the first high-throughput analysis of gut microbiota composition in hybrid grouper-fed BSFL as a partial replacement for FM protein. The data generated from this study could serve as a foundation for formulating grouper feed.

## 2. Materials and Methods

### 2.1. Diet Formulation and Preparation

Four isonitrogenous and isocaloric diets were developed to examine the effects of feeds supplemented with BSFL (Table 1). These diets were formulated to meet the protein and energy requirements of juvenile groupers. A basal diet without BSFL meal was used as the control group (CK). Three experimental diets were developed by substituting FM with BSFL meal at different percentages (10, 30, and 50%) and labeled as BSFL10, BSFL30, and BSFL50, respectively.

Hybrid groupers with an average weight of 56.49 g ± 0.34 were obtained from a commercial hatchery in Hainan, China. The fish were fed their experimental diets at 08:00 and 16:00 every day for 42 days after 14 days of acclimatization period (fed with CK diet). Water quality parameters, including temperature (30.15 ± 0.5 °C), dissolved oxygen (7.20 ± 0.3 mg L^−1^), salinity (29.75 ± 0.5‰), pH (7.15 ± 0.24), and total ammonia (0.3 ± 0.2 mg L^−1^), were monitored and maintained daily. Before conducting the trial, all animal studies were approved by the Committee for the Care and Use of Creatures at Hainan Tropical Ocean University and Hainan Key Laboratory for Conservation and Exploitation of Tropical Marine Fisheries Resources (code: 2019-1134-A1).

### 2.2. Analysis of Digestive Enzyme Activity in the Intestine

Following a 24 h fasting period at the end of the feeding trial, five fish from each tank were anesthetized with MS-222 (50 mg L^−1^) and placed on an ice tray for dissection. The anterior intestinal tract was collected and flash-frozen in liquid nitrogen before further analysis. The weights of these tissues were recorded before determining enzymatic activity. The tissues were homogenized at 3500 rpm with pre-cooled saline at a ratio of 1:9 (*w*/*v*) at 4 °C for 10 min. The supernatant of the intestinal homogenate was used to evaluate the activity of digestive enzymes according to the manufacturer’s instructions (Trypsin assay kit (A080-2-2), Pepsin assay kit (A080-1-1), Amylase (AMS) test kit (C016-2-1), and Lipase assay kit (A054-2-1), Nanjing Jiancheng Bioengineering Institute, Nanjing, China). Amylase activity was determined as the enzymatic activity required to hydrolyze 10 mg of starch per mg of tissue protein after reaction with the substrate at 37 °C for 30 min. Lipase activity was determined as the amount of 1 µmol of substrate consumed per unit of enzyme activity when 1 g of tissue protein reacts with the substrate for 1 min at 37 °C. Trypsin activity was measured as a 0.003 absorbance change per minute induced by trypsin in 1 mg of tissue protein at 37 °C and pH 8.0. Each unit of pepsin activity was defined as 1 μg of amino acid produced from egg white at 37 °C per mg of tissue protein per minute.

### 2.3. Histopathological Analyses of Fish Intestinal Tissues

Ten fish were randomly selected from each tank after the feeding trial. Intestinal tract tissues were freshly fixed with 4% paraformaldehyde. Standard histological techniques were used to prepare the paraformaldehyde-fixed tissues, which were subsequently embedded in paraffin. The samples were sectioned to a thickness of 5 μm and mounted on glass slides. The slides were stained with hematoxylin and eosin (H&E) and examined using light microscopy at 40× and 100× magnifications. Image-Pro Plus 6.0 software (Media Cybernetics, Rockville, MD, USA) was used to evaluate various aspects of the intestinal tissue, such as muscular thickness, villi length, villi width, intestinal wall thickness, and mucosal thickness.

### 2.4. Intestinal Content Collection

Following a 6-week feeding trial, five hybrid groupers were randomly selected from the CK, BSFL10, BSFL30, and BSFL50 groups. The fish were euthanized using MS-222 before dissection. The intestinal contents of each fish were carefully squeezed out and harvested separately under aseptic conditions. All 20 samples were immediately frozen at −80 °C until DNA extraction. High-throughput sequencing and analysis of the 16S rRNA gene V4-V5 region were used to characterize the bacterial communities in the gastrointestinal contents of the 20 fish.

### 2.5. Extraction of Bacterial DNA

The bacterial DNA was extracted from the samples using the PowerDNA Isolation Kit (MoBio, Carlsbad, CA, USA) according to the manufacturer’s instructions. DNA purity and concentration were evaluated using a NanoDrop spectrophotometer (Thermo, Waltham, MA, USA) and a Qubit fluorometer (Thermo, USA). The bacterial community structure was characterized by targeting the 16S rRNA V3 and V4 hypervariable regions with a primer set (341F: 5′-CCT AYG GGR BGC ASC AG-3′; 806R: 5′-GGA CTA CHV GGT WTC TAA T-3′) containing Illumina 3′ adaptor sequences and a 12 bp barcode. Sequencing was performed with an Illumina HiSeq2500 sequencer at Shenzhen Huitong Biotechnology Co., Ltd. (Shenzhen, China).

### 2.6. Amplicon Generation and Library Preparation

Amplicons were generated from 20 to 30 ng of DNA, targeting the V3 and V4 hypervariable regions of bacterial 16S rDNA for subsequent taxonomic analysis. A set of primers was designed to target conserved areas near the V3 and V4 hypervariable regions of the 16S rDNA of bacteria and archaea (forward: 5′-CCT ACG GRR BGC ASC AGK VRV GAA T-3′; reverse: 5′-GGA CTA CNV GGG TWT CTA ATC C-3′). Tagged adaptors were added to the termini of the 16S rDNA amplicons to prepare tagged libraries for downstream NGS sequencing on the Illumina MiSeq sequencer (Illumina, San Diego, CA, USA). PCR reactions were initiated in triplicate using a 25 µL mixture consisting of 2 µL NTP, 2.5 µL Trans Start Buffer, 20 ng DNA template, and 1 µL primer.

### 2.7. Illumina MiSeq Sequencing

The concentration of the DNA libraries was validated using a Qubit 3.0 Fluorometer before sequencing. The DNA libraries were multiplexed and applied to the Illumina MiSeq platform (Illumina, San Diego, California, USA) following the manufacturers’ instructions. Sequencing was performed with the PE250/300 Pairing System, while image analysis and base calling were conducted using the MiSeq Control Software (MCS, v4.0) on the MiSeq instrument(Illumina, San Diego, CA, USA).

### 2.8. Enriched and Amplified DNA Libraries

The metagenomic computational library was constructed following standard Illumina TruSeq DNA library preparation protocols. Barcoded Illumina MiSeq genome sequencing was performed using an Illumina MiSeq Genome Analyzer (Illumina, San Diego, CA, USA). A PE2500 sequencing strategy was employed to create one paired-end (PE) library for each sample, with a PE library insert size of 500 bp. Each sample yielded a minimum of 20,000 pairs of reads, and the quality score of the clean sequencing data was Q30 > 75%. DNA fragments with protruding ends were repaired using a mixture of 3′-5′ exonuclease and polymerase. A unique base A was inserted into the repaired smooth 3′-end of the DNA fragments. At the 3′-end of the junction, a thymine base facilitates complementary pairing with adenine. Selective enrichment of double-ended DNA fragments and simultaneous amplification of DNA libraries were achieved via PCR.

### 2.9. Functional Predictions of the Intestinal Microbiota

PICRUSt (Phylogenetic Investigation of Communities by Reconstructing the Observed State) was employed to predict the functional properties of microbial communities by analyzing the genetic composition of microorganisms [42]. The KEGG database and operational taxonomic units (OTUs) were used to filter collected reads according to a Greengenes reference taxonomy (Greengenes 13.5).

### 2.10. Bioinformatics and Statistical Analyses

Reads from the sequencer were sorted into individual samples based on their unique barcodes. The barcode and primer sequences identified by the main sequence were then removed. Initial rRNA tags were obtained by merging the reads with FLASH (V1.2.7, http://ccb.jhu.edu/software/FLASH/, accessed on 11 August 2020). High-quality tags were filtered using QIIME with the SILVA database (Silva 132, http://qiime.org/index.html, accessed on 11 August 2020) to detect chimera sequences as described by Edgar et al. [43]. The tags were clustered into operational taxonomic units (OTUs) using UPARSE (http://drive5.com/uparse/, accessed on 11 August 2020) at a 97% nucleotide composition similarity [44]. QIIME was subsequently used to determine the phylogenetic placement of selected OTUs. Microbial diversity at the alpha and beta levels was evaluated using Shannon’s and Chao1 indices based on the relative richness of OTUs. Principal component analysis (PCA) measured the relatedness of community composition among samples (beta diversity). Boxplots and heatmaps for selected microbes were generated using the R statistical environment (v3.1.2). Function profiles of the gut microbiome were analyzed with PICRUSt (PICRUSt2, v2.4.1) [42]. 16S rRNA data analysis was performed using the QIIME data analysis package (QIIME 1, http://qiime.org/index-qiime1.html accessed on 11 August 2020).

## 3. Results

### 3.1. Digestive Intestinal Enzyme Activity

Table 2 summarizes the activity of digestive enzymes in the intestine. Among the experimental groups, the fish group fed the BSFL50 diet exhibited the highest pepsin activity, followed by the BSFL30 diet. The lowest activity was observed in fish fed with the CK and BSFL10 diets.

Statistical analyses revealed significant differences (*p* < 0.05) in amylase activity between fish-fed CK, BSFL10, and BSFL50. Fish-fed BSFL50 had the highest lipase activity, followed by BSFL30-fed fish, with the lowest activity in fish-fed BSFL10. Fish-fed BSFL10 exhibited significantly different (*p* < 0.05) trypsin activity compared to the CK group and other BSFL treatments.

### 3.2. Histological and Morphological Analyses of the Intestinal Tissues

**Figure 1:**
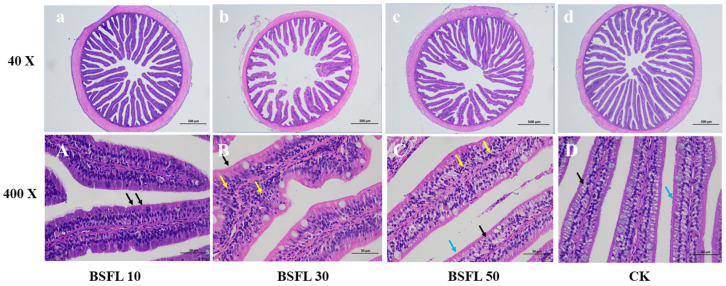
H&E-stained intestinal tissue sections of juvenile hybrid grouper-fed different diets: (**A**,**a**), (**B**,**b**), (**C**,**c**), and (**D**,**d**) are the whole anterior histological sections of the 10%, 30%, 50%, and CK groups, respectively.

Figure 1 shows the histological examination of the intestine. The findings from our examination of intestinal morphology are summarized in Table 2. Substituting 10% of FM with BSFL resulted in a normal intestinal tissue structure, with intact integrity and well-defined striated margins (black arrow, Figure 1A). The intestinal villi were intact and neatly extended into the intestinal lumen, showing no signs of damage or shedding. The muscle thickness, villi length, villi width, intestinal wall thickness, and mucosal thickness of the intestine were comparable between the BSFL10 and CK groups, indicating that the BSFL10 diet did not have any discernible impact on intestinal morphology. The CK group exhibited well-developed intestinal villi, with closely organized columnar epithelial cells. The cytoplasm appeared loose and faintly stained, exhibiting foamy characteristics (black arrow, Figure 1D), with sparse striated margins (blue arrow, Figure 1D). Goblet cells were not prominent in either the CK or BSFL10 groups. When 30% and 50% of FM were substituted with BSFL, the aforementioned measurements appeared shorter and thinner. The villi were sparse, and the intestinal epithelial cells contained vacuoles (black arrow, Figure 1B,C), along with a few striated edges (blue arrows, Figure 1C). Furthermore, there was a slight increase in local subepithelial lymphocytes (yellow arrows, Figure 1B,C), along with an increase in goblet cells.

### 3.3. Illumina Sequencing Quality Analysis and α-Diversity Analysis

**Figure 2:**
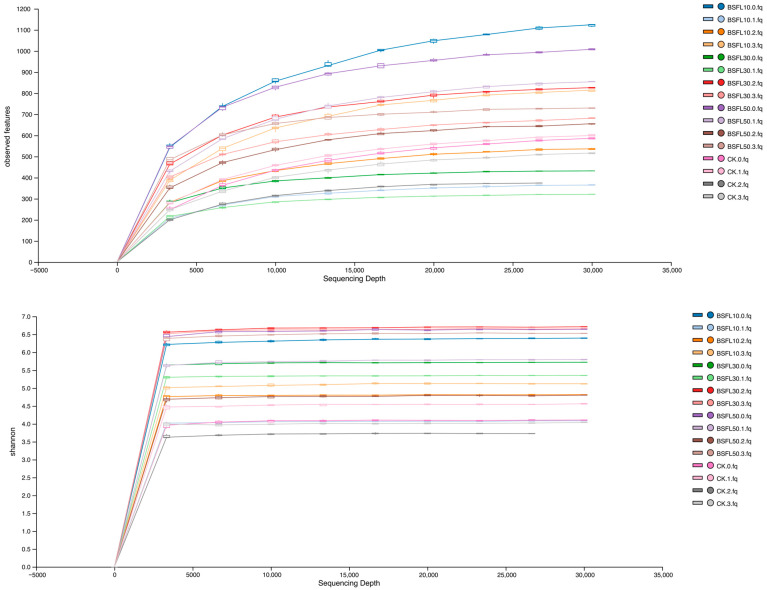
Sobs index curve (**above**) and Shannon index curve (**below**) of the intestinal microbiota of hybrid grouper *(Epinephelus fuscoguttatus* ♀ × *E. lanceolatus* ♂).

A total of 318,928 valid sequences were obtained from 16 samples through high-throughput sequencing. The number of sequences per sample ranged from 30,529 to 50,996, with an average of 39,866 sequences. Each treatment had a coverage rate above 0.99, indicating that the OTUs of the samples sufficiently represented their diversity (Table 3). The samples were normalized to 30,000 sequences before subsequent α-diversity and β-diversity analyses to minimize potential errors in diversity analysis due to variations in sequencing depths among samples. Analysis of the rarefaction curve (Figure 2) revealed a consistent trend as sequencing volume increased, indicating that the Sobs or Shannon index of each sample did not increase further with additional sequencing data. All samples were fully sequenced, encompassing a broad range of species.

The results of the Sobs, Chao, Shannon, Simpson, and Coverage indices representing the intestinal microflora of groupers are displayed in Table 4. All treatment groups demonstrated high coverage. The Sobs index was significantly higher in the BSFL-treated groups compared to the CK group (*p* < 0.05), with BSFL10 and BSFL50 showing significantly greater values than CK. The trend in the gut microbiota abundance index (Chao) correlated with that of the Sobs index. The Shannon index was significantly higher in the BSFL groups compared to the CK group (*p* < 0.05), with the BSFL10 and BSFL50 groups having values 48.85% and 44.63% higher than the CK group, respectively. Variability in the Simpson index was significantly greater in the BSFL30 group compared to the CK group (*p* < 0.05), with no significant difference observed between the BSFL30 group and the other groups. These results indicate that BSFL can enhance the abundance and diversity of intestinal flora after replacing fishmeal at various levels.

### 3.4. Distribution of Intestinal Microbiota

**Figure 3:**
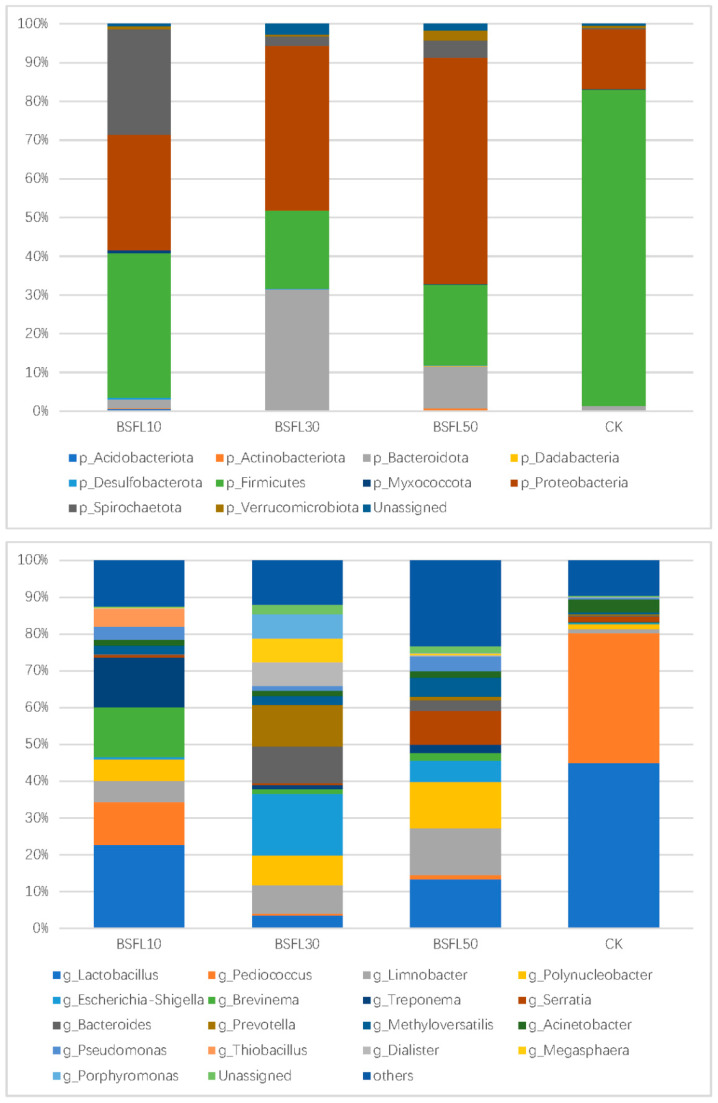
The bacterial community structure of intestinal microbiota of hybrid grouper (*Epinephelus fuscoguttatus* ♀ × *E. lanceolatus* ♂) at phylum (**upper**) and genus levels (**below**).

Figure 3 shows the structure of the microbial community found in the intestines of groupers. The dominant bacteria at the phylum level were Firmicutes, Proteobacteria, Bacteroidetes, Spirochaetota, and Verrucomicrobia, with Firmicutes, Proteobacteria, and Bacteroidetes exhibiting the highest prevalence. In the CK group, Firmicutes were 2.53, 12.03, and 5.87 times more abundant than in the BSFL10, BSFL30, and BSFL50 groups, respectively. The BSFL replacement groups exhibited higher levels of Proteobacteria, Bacteroidetes, and Spirochaetota compared to the CK group. Proteobacteria were significantly less abundant in the CK group compared to the BSFL10, BSFL30, and BSFL50 groups by 68.33%, 59.05%, and 153.90%, respectively. Bacteroidetes and Spirochaetota were most abundant in the BSFL30 group, followed by the BSFL50 group, while the CK group had the lowest abundance.

At the genus level, *Lactobacillus* and *Pediococcus* were the most prevalent. The CK group had higher levels of *Lactobacillus* and *Pediococcus* compared to the BSFL replacement group. The abundance of *Lactobacillus* in the CK group was 2.29, 21.49, and 4.97 times higher than in the BSFL10, BSFL30, and BSFL50 groups, respectively. The abundance of *Pediococcus* in the CK group was 3.49, 153.89, and 52.26 times higher than in the BSFL10, BSFL30, and BSFL50 groups, respectively. Other highly abundant bacteria included *Limnobacter*, *Polynucleobacter*, *Escherichia–Shigella*, *Brevinema*, *Treponema*, *Bacteroides*, *Prevotella*, *Methyloversatilis*, *Acinetobacter*, and *Pseudomonas*. The group that had FM substituted with BSFL exhibited significant increases compared to the CK group, indicating that substituting FM with BSFL in the formulated diets altered the bacterial community structure of groupers at the phylum and genus levels.

### 3.5. Changes in the Microbial Community Structure

**Figure 4:**
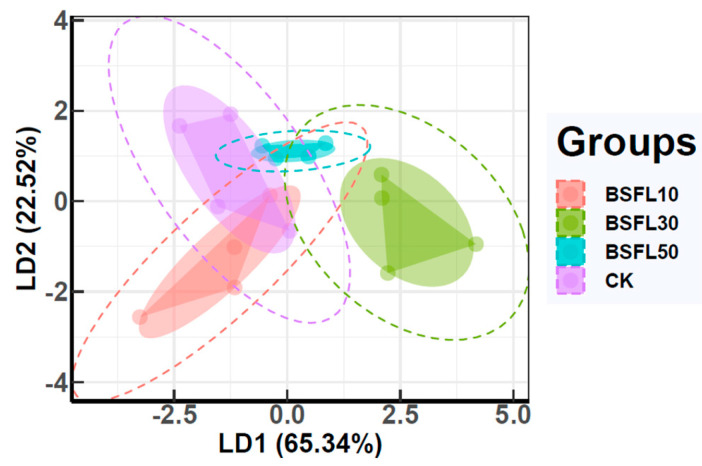
Linear discriminant analysis (LDA) of intestinal microbiota in different groups of hybrid grouper (*Epinephelus fuscoguttatus* ♀ × *E. lanceolatus* ♂).

Linear discriminant analysis (LDA) is a generalized version of Fisher’s linear discriminant approach used to identify a linear combination of features from two classes of objects or events for characterization or discrimination. As shown in Figure 4, LD1 and LD2 accounted for 65.34% and 22.52% of the variance, respectively. BSFL30 showed distinct differences from the CK group as well as from BSFL10 and BSFL50. In contrast, BSFL10 appeared to be closer to the CK group. There was a small overlap between the BSFL50 and CK groups, suggesting a considerable alteration in the intestinal microflora structure in the BSFL replacement groups compared to the CK group. The BSFL30 group exhibited the most significant changes. The partial overlap between the BSFL10 group and CK group may have resulted from the restoration of the intestinal microflora structure as the grouper adapted to the increased level of BSFL substitution.

### 3.6. Screening of Taxonomic Units and Heatmap Analysis

**Figure 5:**
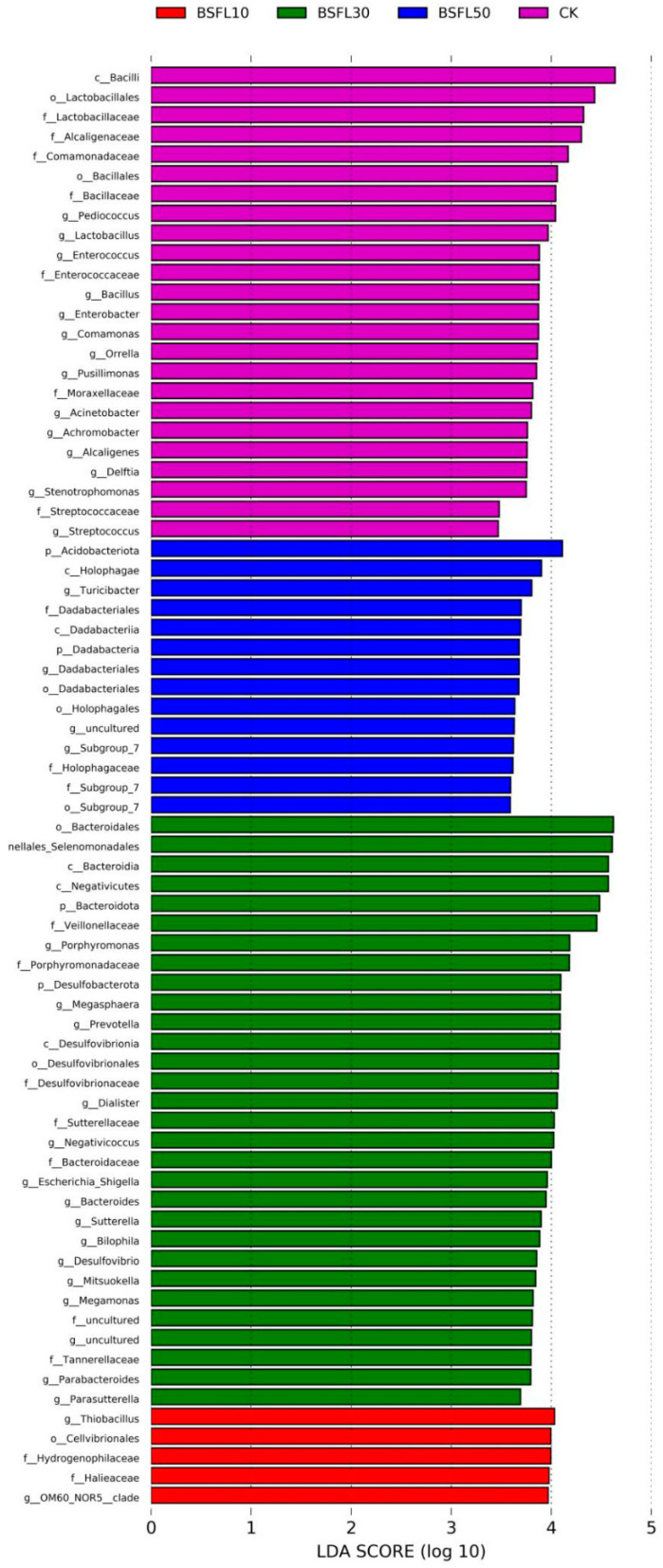
The LDA scores of intestinal microbiotas with different abundances in different groups of hybrid grouper (*Epinephelus fuscoguttatus* ♀ × *E. lanceolatus* ♂) at the genus level.

**Figure 6:**
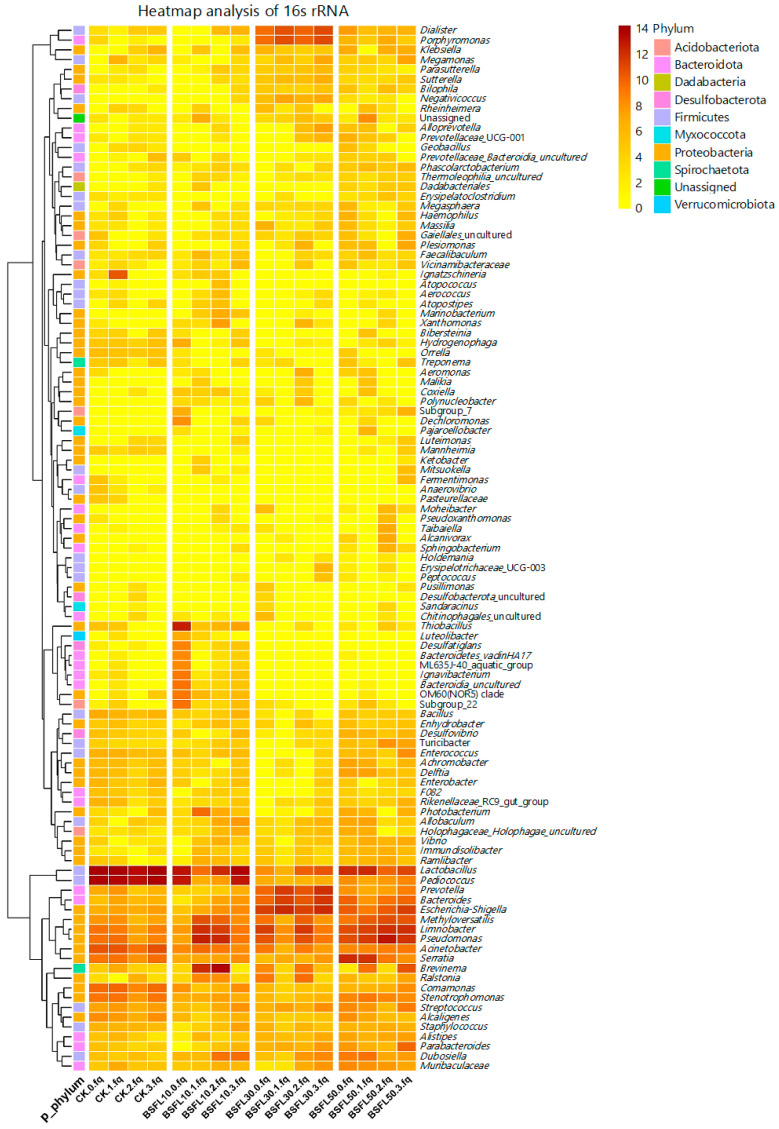
Heatmap of intestinal microbiota in different groups of hybrid grouper (*Epinephelus fuscoguttatus* ♀ × *E. lanceolatus* ♂) at the genus level.

The use of linear discriminant analysis effect size (LEfSe) revealed significant differences in bacterial abundance at the genus level between groupers-fed BSFL-substituted feeds and the CK group (Figure 5). Differences were determined using LDA, with a score threshold greater than 3.5. The length of the histogram corresponds to the magnitude of the effect of the various species (LDA score). In Figure 5, the CK group showed a predominance of *Bacilli*, *Lactobacillaceae*, *Alcaligenaceae*, *Comamonadaceae*, *Bacillalles*, *Pediococcus*, and *Lactobacillus*. In the BSFL10 group, *Thiobacillus*, *Cellvibrionales*, *Hydrogenophilaceae*, and *Halieaceae* were the prominent taxa. The major taxa in the BSFL30 group were *Bacteroidales*, *Selenomonadales*, *Negativicutes*, *Bacteroidota*, *Veillonellaceae*, and *Porphyromonadaceae*. The most significant taxa in BSFL50 were *Acidobacteriota*, *Holophagae*, *Turicibacter*, *Dadabacteriales*, *Dadabacteriia*, *Dadacacteria*, and *Dadabacteriales*. The heatmap analysis revealed that the grouper gut microbiota was primarily dominated by *Acidobacteriota*, *Bacteroidota*, and *Dadabacteria* when the fish were fed diets containing BSFL (Figure 6). As the levels of BSFL in the diet increased, genera such as *Dialister*, *Porphyromonas*, *Bilophila*, *Negativicoccus*, *Alcanivorax*, and *Holdemania* became more prominent.

**Figure 7:**
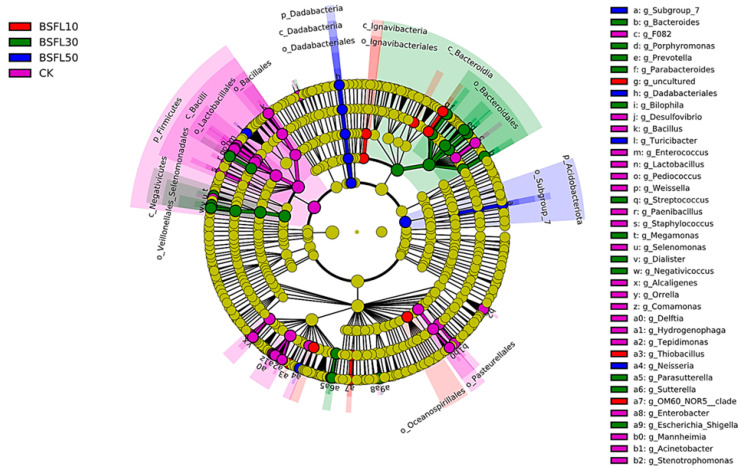
The evolutionary clade diagram of intestinal microbiota in different groups of hybrid grouper (*Epinephelus fuscoguttatus* ♀ × *E. lanceolatus* ♂).

**Figure 8:**
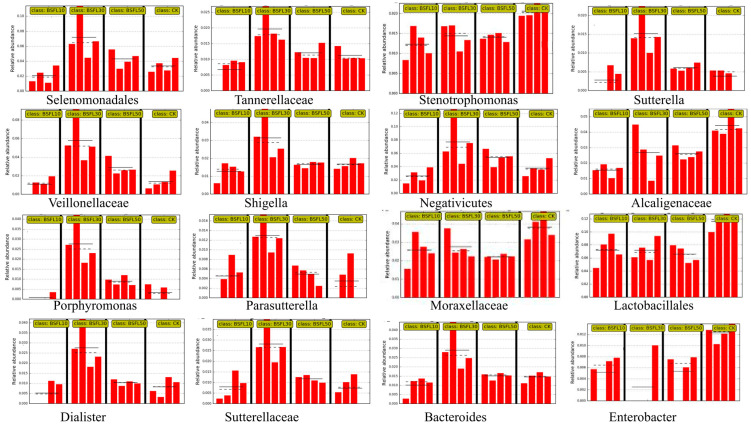

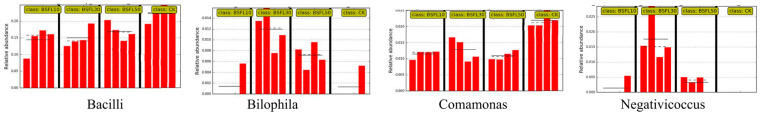
Abundance of significantly different species of intestinal microbiota in various groups of hybrid grouper (*Epinephelus fuscoguttatus* ♀ × *E. lanceolatus* ♂).

Figure 7 illustrates the multilevel species hierarchy tree derived from the LEfSe analysis. The circle radiating outward represents the phylum-to-genus classification level (or species), with the innermost yellow circle marking the border. A total of 58 genera of bacteria were identified as significant factors in distinguishing between the groups. The BSFL10, BSFL30, and BSFL50 groups exhibited a similar in comparison to the CK group, with relative abundances greater than 0.01%. This suggests significant differences in abundance (Figure 8). The CK group had slightly higher relative abundances of *Moraxellaceae*, *Lactobacillus*, *Enterobacter*, *Bacillaceae*, *Comamonas*, *Stenotrophomonas*, and *Alcaligenes* compared to the BSFL10, BSFL30, and BSFL50 groups. Furthermore, the BSFL30 and BSFL50 groups showed significantly higher relative abundances of various bacterial genera including *Porphyromonas*, *Parasutterella*, *Dialister*, *Sutterellaceae*, *Bacteroides*, *Bilophila*, *Negativicoccus*, *Selenomonadales*, *Tannerellaceae*, *Sutterella*, *Veillonellaceae*, *Shigella*, and *Negativicutes*.

### 3.7. Prediction of Metabolic Function

**Figure 9:**
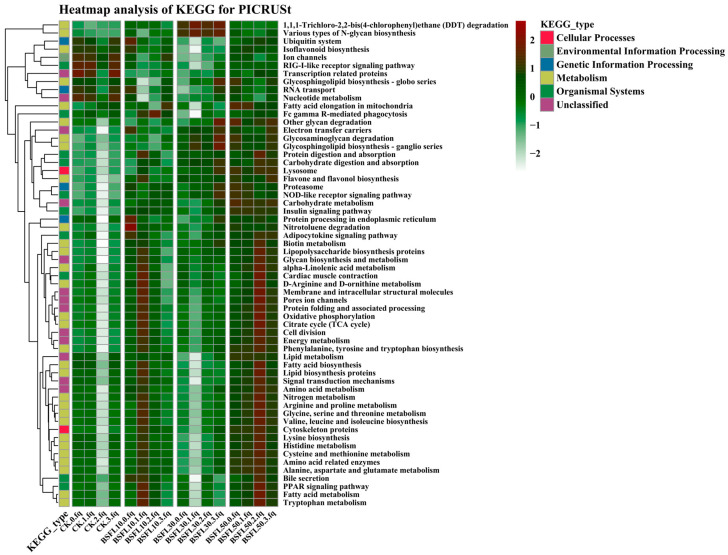
Prediction of intestinal microbiota functional pathway in different groups of hybrid grouper (*Epinephelus fuscoguttatus* ♀ × *E. lanceolatus* ♂).

PICRUSt analyses were performed to predict gene functions by assessing the microbial gene composition (Figure 9). This analysis identified 262 gene families through the clustering of KOs (KEGG Orthology). Level 3 KEGG pathway analysis revealed that genes associated with metabolism (e.g., fatty acid elongation in mitochondria and glycan degradation), electron transfer carriers, carbohydrate metabolism, and glycan biosynthesis were significantly lower in the CK group compared to the BSFL replacement group. Microbial genes in the intestines of the BSFL10 and BSFL50 treatment groups were associated with nutritional metabolism, including amino acid, nitrogen, and fatty acid metabolism. Furthermore, compared to the CK group, uncategorized clusters related to membrane and intracellular structural molecules, porous channels, protein folding and processing, cell division, signal transduction mechanisms, and cellular processes such as lysosome and cytoskeleton proteins exhibited a similar trend.

## 4. Discussion

The hybrid grouper diet primarily consists of a mix of FM and plant-based feed, such as soybean meal [45,46]. Previous studies have explored the effects of replacing FM with soybean meal in these fish and found that the grouper’s ability to utilize soy protein concentrate (SPC) as a dietary protein source is limited [41]. It has been recommended to restrict fishmeal replacement to below 30% (FM 45.5 g 100 g^−1^ and SPC 18 g 100 g^−1^) [41]. Similar results were observed in studies involving other fish species, such as Siberian sturgeon [19], rainbow trout [47,48], African catfish [49], European seabass [33], and juvenile Jian carp [50].

Literature has emphasized the importance of gut microecology in understanding the role of nutrients and their metabolism in fish nutrition [8,9]. This study is the first to investigate the effects of substituting FM with BFSL in diet formulations for hybrid grouper using a comprehensive analysis of intestinal histopathology and microbial composition. The fish’s intestinal tract is the anatomical basis for nutrient digestion and absorption. Its histological composition includes the mucosa, submucosa, muscularis, and plasma. Within the mucosa, columnar epithelial cells aid in absorption, while goblet cells produce digestive enzymes and mucus. These components are crucial for maintaining structural integrity, promoting digestion, regulating microbiome balance, and supporting immune function [51]. Therefore, maintaining the integrity of the intestinal structure is vital for the digestive capabilities of fish. Furthermore, impaired intestinal integrity can lead to pathogenic breaches in the epithelium, causing immune cell infiltration and inflammatory responses [52].

The present study revealed that the diets had no significant impact on intestinal amylase activity. However, intestinal lipase activity in the grouper increased with higher supplementation of BSFL in the diet, particularly in the BSFL50 group. This finding is consistent with a previous study on rice field eels (*Monopterus albus*) fed with BSFL (15.78%). That study demonstrated that substituting BSFL below 15.78% improved growth and balanced intestinal flora in the fish, but higher levels negatively impacted lipid metabolism [53].

Interestingly, lipase serves as a signaling molecule that regulates lipid metabolic pathways [54]. The levels of pepsin and trypsin were significantly higher in the BSFL50 and BSFL10 groups compared to the control group. Moreover, increasing the amount of BSFL (30% and 50%) in the FM led to a significant reduction in various intestinal parameters of the fish, such as muscle thickness, villi length, villi width, wall thickness, and mucosal thickness.

In this present study, vacuoles and thinner intestinal walls were observed at 30–50% BSFL. This may explain the differences in digestive enzyme activity observed among the treatment groups. In [55], it was reported that the presence of chitin in BSFL might trigger an inflammatory response in the intestine, potentially compromising tissue integrity. However, previous studies utilizing insect-based diets (*Hermetia illucens*) with 25% and 50% FM substitution in zebrafish (*Danio rerio*) did not reveal any detrimental effects on the intestinal tissues of the zebrafish [56]. In cork fish (*Channa striata*), higher concentrations of BSFL (20%, 50%, 80%, and 100%) had no significant impact on growth but did increase the levels of lipase, amylase, and protease enzymes [57].Crude protein levels in fish diets are often overestimated; however, insect meals can contain significant amounts of non-protein nitrogen. In fact, non-protein nitrogen, such as chitin, makes up about 16–30% of the total nitrogen in BSFL meal [58,59]. Chitin is a nitrogenous polymer found in insect cuticles and is considered a non-protein source of nitrogen [18,60]. It is important to note that chitin is indigestible for fish and is generally classified as an antinutrient [61]. A study by Guerreiro et al. [62] found that diets for meagre fish, *Argyrosomus regius*, particularly those with high chitin content from BSFL can reduce nutrient digestibility [60]. Since chitin is a primary component of insect bodies, it cannot be eliminated from the diet when using BSFL as a raw material [63]. Guerreiro et al. (2021) [62] stated that fish can digest chitin in the presence of the chitinase enzyme found in their stomachs and intestines. In our study, since we did not specifically assess the chitin content in the feed or the levels of chitinase enzyme in the stomachs and intestines of grouper, it is insufficient to evaluate the role of chitin. In the future, it is worthwhile to conduct related research to address this issue.

The results of this study demonstrate a significant difference in the diversity and abundance of intestinal microbes between the BSFL and CK groups [64,65]. In our study, we observed a 6-week (excluding a 2-week acclimatization period) dietary modulation of intestinal bacterial communities, and the method of collecting the intestinal sample is consistent with the literature (Nayak, 2010a, 2010b; Loh et al., 2021) [3,66,67]. This allowed us to assess the effects of diet on the gut microbiome at the same point in time, ensuring consistency and comparability of results. Although different effects on gut microbiome modulation were reported, the results are still inconclusive. To the best of our knowledge, no data have been reported regarding the effect of BSFL on the intestinal microbiome in hybrid grouper (*Epinephelus fuscoguttatus* ♀ × *E. lanceolatus* ♂). Previous studies have shown that bacteria from the *Firmicutes*, *Proteobacteria*, *Bacteroidetes*, *Spirochaetota*, and *Verrucomicrobia* phyla are abundant in fish intestines [8,40,66,68,69,70,71]. The CK group in the present study had a higher abundance of Firmicutes compared to the BSFL group, while the BSFL group was predominantly composed of Proteobacteria, Bacteroidetes, and Spirochaetes. Studies have also shown that microorganisms from the Firmicutes and Bacteroidetes phyla can enhance fish digestive and immunological health by combating pathogenic bacteria [72,73,74]. Proteobacteria and Firmicutes are commonly found in the intestinal microbiota of salmon, indicating their adaptation to fish intestinal environments or aquatic conditions [75,76]. The presence of Spirochaetota in the BSFL group is noteworthy, as these bacteria play a crucial role in gut microbiome homeostasis, as demonstrated in a study on *L. crocea* [77]. Defosse et al. [78] discovered the spirochete *Brevinema andersonii*, which can be isolated from autophagosomes. In a study on *Trachinotus ovatus*, it was observed that these spirochaete bacteria significantly increased in diseased fish, along with a notable decrease in the levels of Firmicutes and Bacteroidetes compared to healthy fish [79]. Wang et al. [80] found that seahorses (*Hippocampus kuda*) with enteritis had elevated levels of *Spirochaeta*, *Mycobacterium*, and *Vibrio* bacteria. However, the grouper samples in our study did not show signs of illness and exhibited normal feeding behavior. The nature of the *Spirochaeta* bacteria in the intestines remains unknown and requires further investigation. Furthermore, beneficial bacteria groups such as *Lactobacillus*, *Lactococcus*, *Bacillus*, and *Saccharomyces* species were identified in the BSFL group. These findings explain the increased digestive enzyme activity in the BSFL group compared to the CK group in our study. Moreover, *Lactobacillus*, *Leuconostoc*, *Lactococcus*, *Enterococcus*, *Shewanella*, *Carnobacterium*, *Aeromonas*, *Vibrio*, *Bacillus*, *Enterobacter*, *Pseudomonas*, *Clostridium*, and *Saccharomyces* species are common probiotics used in aquaculture practices [72,81,82,83].

The role of microbiota in promoting fish health, disease resistance, and other beneficial functions has been extensively documented. This study showed that including optimal levels of insect meal in fish diets can benefit hybrid grouper aquaculture by modifying the gut microbiota. The main groups in *Firmicutes* are *Clostridia* and *Bacillaceae*, while *Aeromonas* and *Enterobacteriaceae* are commonly found in *Proteobacteria*. *Enterobacteriaceae* makes up 50% of all bacteria and is frequently present in the gut flora of farmed fish-fed artificial diets [84]. High levels of *Enterobacteriaceae* have been observed in the intestinal chyme of fish-fed *H. illucens* diets [19,74]. These findings are consistent with the study by Borrelli et al. [85], which showed that *H. illucens* significantly enhances the diversity of microbial populations. Additionally, *Enterobacteriaceae* bacteria are considered normal in fish farmed near human populations [86,87,88]. Józefiak et al. [19] further confirmed that incorporating full-fat insect meal into feed can stimulate the proliferation of lactic acid bacteria (LAB) such as *Lactobacillus* and *Enterococcus* spp. in rainbow trout. LAB adherence to the intestinal mucosa can improve mucosal function and morphological development in rainbow trout [89]. LABs are commonly used as probiotics in fish to enhance immune responses, protect against infections, and improve nutritional absorption [66,67]. The immunomodulatory effect may be attributed to chitosan derived from *H. illucens* and its oligosaccharide derivatives (chitosaccharides). Chitin is recognized as a prebiotic that can enhance intestinal absorption [15,90,91] and promote the growth of beneficial bacteria while inhibiting harmful pathogens, fungi, and viruses [91,92]. Additionally, chitin reduces oxidative stress in fish [91] and facilitates the colonization of beneficial gut flora. Chitin found in insect exoskeletons exhibits antioxidant, antifungal, and antiviral properties [93,94]. Interestingly, a recent study demonstrated that *H. illucens* possesses a wide range of genes, such as glutathione S-transferase 1 and UDP-glucosyltransferase 2, that can effectively metabolize aflatoxin B1. This metabolic capability plays a critical role in preventing the contamination of mycotoxins in feed [95].

Earlier studies have identified the genus *Pseudomonas* as a natural part of the microbiome of seabass and Gulf killifish, supporting our findings [96,97,98]. In this study, we found that the genus *Pseudomonas* was particularly abundant and showed a high replacement of BSFL. However, more research is needed to fully understand this phenomenon. *Pseudomonas* is an opportunistic pathogen that can produce various antibiotics and small bioactive molecules under favorable conditions, exhibiting antibacterial lysozyme effects. In adverse conditions, it can cause surface ulceration, swelling, and congestion in fish intestines, as observed in fish-fed diets with high levels of BSFL substitution (30% and 50%).

Previous studies have shown that *Brevinema andersonii* and another member of the *Spirochaetota* phylum are more common in the mucosal layer of the terminal intestine than in the chyme [22]. In our study, we found common spirulina in hybrid groupers-fed diets with high levels of BSFL substitution (30% and 50%). Recent research indicates that *B. andersonii* is more prevalent in the gut mucosa than in the digesta of seawater-phase Atlantic salmon, regardless of dietary intake [99]. Spirochetes are known for their high motility and chemotaxis toward mucins. Some species can penetrate mucus and attach to the gut mucosa [100,101]. However, further studies are needed to determine if this taxon accumulates in the layer of intestinal mucus regardless of dietary composition. The abundance of *Dadabacteria* in the intestines of fish fed the high-BSFL alternative diet was greater than that in fish fed other diets. Marine *Dadabacteria* are believed to be widespread in the marine domain and represent a potential phylum. However, a comprehensive evaluation of the available genome data for this bacterium is lacking, hindering our understanding of its genomic structure in the marine environment. Graham and Tully [102] found that upper mid-ocean *Dadabacteria* genomes are streamlined, with shorter genomes and reduced nitrogen content in DNA and projected proteome compared to their phylogenetic counterparts. The authors propose that *Dadabacteria* can break down microorganisms that degrade organic substances, particularly peptidoglycans and phospholipids.

Various factors, such as diet, phylogeny, and the development of the core microbiome, influence the fish microbiome. In a study by Zeng et al. [103], variations in the gut flora of four different feeding fish species (i.e., *Hypophthalmichthys molitrix*, *Ctenopharyngodon idellus*, *Aristichthys nobilis*, and *Carassius auratus*) were observed. This suggests that bacteria from sources other than plankton may significantly impact the taxonomic profile of fish. The physicochemical parameters of the aquatic environment, including temperature, salinity, pH, oxygen levels, pollutants, seasonality, nutrient biorhythms (food supply), antimicrobials, and other factors, directly affect the gut microbiota [104,105,106,107,108].

In our previous unpublished data, substituting less than 20% of BSFL had beneficial effects on the digestive capacity and immunological response of hybrid grouper. The present study analyzed the structure of the intestinal flora, identifying significant differences in abundance among taxonomic groups and predicting their metabolic processes. This allowed for the investigation of the biological significance of substituting FM with BSFL in regulating the intestinal microbiota of grouper. Investigating the functional diversity of bacterial communities emphasizes the importance of the gut microbiome. The analysis of KEGG orthologs (KOs) linked to the intestinal microbial function in the four dietary groups indicated that the primary gene functions of identified bacteria were associated with metabolic processes, consistent with previous findings [109,110]. In the present study, genes related to genetic information processing, transcription-related proteins, and nucleotide metabolism were significantly enriched in the CK group. Level 3 KEGG pathway analysis revealed that microbial functional genes involved in the biosynthesis of secondary metabolites, carbohydrate and lipid metabolism, and amino acid metabolism were relatively less abundant in the CK group but significantly more abundant in the BSFL-replacement groups. Fish fed with the BSFL10 and BSFL50 diets exhibited a clustering of large microbial genes in the intestine, primarily associated with energy metabolism, amino acid metabolism, nitrogen metabolism, and lipid metabolism. Furthermore, genes related to cellular formation and processes, signal transduction mechanisms, and genetic information processing were highly expressed in the intestines of fish fed with BSFL10 and BSFL50 diets compared to the CK group, indicating potential involvement in redesigning the microbial membrane structure [111,112].

Previous studies have shown differences in microbial community function across various regions of the intestine [113]. For instance, in grass carp, microorganisms in the hindgut exhibit higher energy and carbohydrate metabolism compared to those in the foregut [114]. This study confirmed similar findings in hybrid grouper and aligns with observations of digestive enzyme activity and histological examination of the intestine.

## 5. Conclusions

This study demonstrates the potential of replacing fishmeal (FM) with black soldier fly larvae (BSFL) in marine finfish aquaculture, based on identified differences in the intestinal microbiota of grouper fish-fed diets with varying levels of BSFL and FM. The dominant microbiota in different dietary groups plays a critical role in regulating microbial population dynamics and key functional properties related to microbial enzyme production and nutrient metabolism in the intestine. Specifically, our findings suggest that replacing 10% of FM with BSFL could enhance protein, carbohydrate, and lipid metabolism, as well as improve the diversity of the intestinal microbiota in hybrid grouper (*E. fuscoguttatus* ♀ × *E. lanceolatus* ♂). However, further research is needed to explore other factors that may impact the efficacy of replacing FM with insect meal in aquafeed to promote a more sustainable and environmentally friendly aquaculture industry.

## Figures and Tables

**Table 1:** Ingredients and proximate compositions of the experimental diets (as dry-matter basis %).

Ingredients (g 100g^−1^)	Diets			
	CK	BSFL 10	BSFL 30	BSFL 50
Fishmeal (57%) ^a^	40.0	36.0	28.0	20.0
BSFL (35%) ^b^		7.43	22.0	37.1
Soy protein concentrate (65%)	15.0	15.0	15.0	15.0
Casein	5.0	5.0	5.0	5.0
Shrimp meal	2.5	2.5	2.5	2.5
Wheat flour	16.4	15.27	12.9	9.40
Binding agents	3.0	3.0	3.0	3.0
Microcrystalline cellulose	2.0	2.0	2.0	2.0
Soybean oil	1.0	1.0	1.0	1.0
Fish oil	10.1	7.8	3.6	0
Squid visceral ointment	1.5	1.5	1.5	1.5
Vitamin premix ^c^	1.0	1.0	1.0	1.0
Mineral premix ^d^	1.0	1.0	1.0	1.0
Choline chloride	0.5	0.5	0.5	0.5
Monocalcium phosphate	1.0	1.0	1.0	1.0
Proximate composition				
Crude protein	36.72	36.45	37.78	37.75
Crude lipid	14.96	14.67	14.88	14.72
Gross energy (kJ g^−1^)	17.21	17.27	17.34	17.17

^a^ Fish meal: crude protein 57.0%, crude lipid 6.1%, crude fiber 8.4%, crude ash 16.06%, Lys 3.19%, Arg 2.89%, His 2.16%, Asp 6.28%, Glu 9.05%, Gly 4.18%, Ala 4.29%, Val 2.41%, Leu 5.21%, Ile 2.40%, Phe 2.95%, Pro 2.37%, Trp 0.78%, Tyr 1.06%, Ser 2.66%, Met 1.62%, Thr 3.09%. ^b^ Black soldier fly larvae: crude protein 35.0%, crude lipid 32%, crude ash 14.6%, chitin 4.93%, Lys 2.73%, Arg 2.31%, His 0.97%, Asp 3.75%, Glu 5.65%, Gly 3.18%, Ala 3.29%, Val 2.41%, Leu 3.21%, Ile 1.64%, Phe 1.65%, Pro 2.37%, Trp 0.78%, Tyr 2.06%, Ser 1.66%, Met 0.56%, Thr 1.49%. ^c^ Vitamin mixture (mg kg^−1^ diet): retinol acetate, 38.0; cholecalciferol, 13.2; α-tocopherol, 210.0; thiamin, 115.0; riboflavin, 380.0 pyridoxine 88.0; pantothenic acid, 368.0; niacin, 1030.0 biotin, 10.0; folic acid, 20.0; vitamin B12, 1.3; inositol, 4000.0; ascorbic acid, 500.0 (Ding et al., 2010) [41]. ^d^ Mineral mixture (mg kg^−1^ diet): MgSO_4_·7H_2_O, 3568.0; NaH_2_PO_4_·2H_2_O, 25,568.0; KCl, 3020.5; KAl (SO_4_)_2_, 8.3; CoCl_2_, 28.0; ZnSO_4_·7H_2_O, 353.0; Ca-lactate, 15,968.0; CuSO_4_·5H_2_O, 9.0; KI, 7.0; MnSO_4_·4H_2_O, 63.1; Na_2_SeO_3_, 1.5; C_6_H_5_O_7_Fe·5H_2_O, 1533.0; NaCl, 100.0; NaF, 4.0 (Ding et al. 2010) [41].

**Table 2:** Enzyme activities of juvenile hybrid grouper-fed different diets.

Items	Groups			
	CK	BSFL 10	BSFL 30	BSFL 50
Intestine				
Pepsin (U/mg protein)	0.30 ± 0.04 ^c^	0.38 ± 0.05 ^c^	0.84 ± 0.11 ^b^	1.17 ± 0.07 ^a^
Amylase (U/mg protein)	0.71 ± 0.30 ^a^	1.29 ± 0.15 ^a^	1.19 ± 0.05 ^a^	0.90 ± 0.18 ^a^
Lipase (U/g)	1.17 ± 0.17 ^b^	1.03 ± 0.17 ^b^	1.41 ± 0.10 ^ab^	2.12 ± 0.30 ^a^
Trypsin (U/mg protein)	2139.07 ± 161.11 ^c^	5904.35 ± 198.88 ^a^	3134.97 ± 201.82 ^b^	1905.98 ± 62.45 ^c^

Values (mean ± SD, n = 5) in the same row with different letters are significantly different (*p* < 0.05). The absence of letters indicates no significant difference between treatments.

**Table 3:** Selection of the valid DNA sequences.

Sample-ID	Raw PE (#)	Combined	Qualified	Nochime (#)	Base (nt)	AvgLen (nt)	Q20	Q30	GC %	Effective %
BSFL10.0	95,058	86,925	84,140	60,221	25,497,648	423	98.15	94.22	53.37	63.35
BSFL10.1	96,464	93,295	91,137	61,637	25,555,748	415	98.38	94.66	53.02	63.9
BSFL10.2	99,033	92,496	90,011	64,678	27,180,916	420	98.04	93.86	52.2	65.31
BSFL10.3	85,341	71,964	69,178	57,014	23,943,945	420	98.23	94.49	53.8	66.81
BSFL30.0	75,879	57,136	55,113	49,231	20,371,855	414	97.52	92.84	52.25	64.88
BSFL30.1	64,755	55,761	54,001	43,067	17,827,745	414	98.27	94.45	50.65	66.51
BSFL30.2	100,928	76,286	71,966	62,162	25,625,892	412	97.73	93.25	51.79	61.59
BSFL30.3	94,196	85,646	82,673	66,766	27,675,346	415	98.16	94.25	52.01	70.88
BSFL50.0	78,874	73,337	71,620	62,314	25,931,448	416	98.36	94.76	54.64	79.10
BSFL50.1	95,222	88,670	86,568	65,211	27,121,028	416	98.29	94.55	54.77	68.48
BSFL50.2	92,718	88,529	86,314	68,622	28,388,865	414	98.38	94.66	53.37	74.01
BSFL50.3	80,809	76,040	74,399	62,482	26,046,974	417	98.24	94.42	53.47	77.32
BSFLCK.0	96,454	88,405	86,070	65,796	27,894,430	424	98.19	94.26	51.62	68.21
BSFLCK.1	86,384	83,556	81,679	61,698	26,141,929	424	98.22	94.36	51.75	71.42
BSFLCK.2	77,818	58,721	51,269	47,730	19,168,573	402	97.41	92.83	51.54	61.34
BSFLCK.3	87,300	80,779	78,574	63,581	26,966,383	424	98.24	94.42	51.63	72.83

**Table 4:** Alpha-diversity index (mean ± SD, n = 3).

Groups	Abundance Index		Diversity Index		Coverage
	Observed Features (Sobs)	Chao	Shannon	Simpson	
BSFL10	706.25 ± 13.95 ^b^	728.00 ± 17.11 ^b^	5.09 ± 0.48 ^b^	0.89 ± 0.03 ^ab^	1.00 ± 0.00
BSFL30	564.00 ± 14.75 ^c^	572.25 ± 18.29 ^c^	6.11 ± 0.35 ^a^	0.95 ± 0.01 ^a^	1.00 ± 0.00
BSFL50	813.25 ± 17.95 ^a^	831.00 ± 19.60 ^a^	5.94 ± 0.42 ^a^	0.91 ± 0.03 ^ab^	1.00 ± 0.00
CK	517.25 ± 11.11 ^c^	534.00 ± 6.71 ^c^	4.11 ± 0.17 ^c^	0.84 ± 0.01 ^b^	1.00 ± 0.00

Values (mean ± SD, n = 3) within the same row with different letters are significantly different (*p* < 0.05). The absence of letters indicates no significant difference between treatments.

## Data Availability

Raw sequences of hybrid grouper gut metagenomic sequencing data were deposited in the Sequence Read Archive (SRA) of the National Center for Biotechnology Information (NCBI) under BioProject accession number PRJNA1113128.
